# Comparative metagenomics reveals the microbial diversity and metabolic potentials in the sediments and surrounding seawaters of Qinhuangdao mariculture area

**DOI:** 10.1371/journal.pone.0234128

**Published:** 2020-06-04

**Authors:** Shuping Wang, Zhenguang Yan, Pengyuan Wang, Xin Zheng, Juntao Fan

**Affiliations:** State Key Laboratory of Environmental Criteria and Risk Assessment, Chinese Research Academy of Environmental Sciences, Beijing, China; Guangzhou University, CHINA

## Abstract

Qinhuangdao coastal area is an important mariculture area in North China. Microbial communities play an important role in driving biogeochemical cycle and energy flow. It is necessary to identify the microbial communities and their functions in the coastal mariculture area of Qinhuangdao. In this study, the microbial community compositions and their metabolic potentials in the sediments and their surrounding seawaters of Qinhuangdao mariculture area were uncovered by the 16S rRNA gene amplicon sequencing and metagenomic shotgun sequencing approaches. The results of amplicon sequencing showed that Gammaproteobacteria and Alphaproteobacteria were predominant classes. Our datasets showed a clear shift in microbial taxonomic groups and the metabolic pathways in the sediments and surrounding seawaters. Metagenomic analysis showed that purine metabolism, ABC transporters, and pyrimidine metabolism were the most abundant pathways. Genes related to two-component system, TCA cycle and nitrogen metabolism exhibited higher abundance in sediments compared with those in seawaters. The presence of cadmium-resistant genes and ABC transporters suggested the ability of microorganisms to resist the toxicity of cadmium. In summary, this study provides comprehensive and significant differential signatures in the microbial community and metabolic pathways in Qinhuangdao mariculture area, and can develop effective microbial indicators to monitor mariculture area in the future.

## Introduction

The microbial communities are widely distributed, and they play a vital role in marine ecosystems. The functions of microbial communities in marine environment balance various important biogeochemical cycles, including degradation of pollutants, nutrient cycles, and organic mineralization [1,2]. To date, ecosystem monitoring technologies are based on the monitoring of total biomass of microorganisms [3], algae [4,5], and macrozoobenthic species [6]. Studies have shown that the microbial diversity and metabolisms depend on the local environment, and are rapid and sensitive to environmental changes [7–9]. Therefore, responses of microbial communities associated with environmental changes can be used as microbial “indicators”. The identification of the core microbiome of a given environment is critical to define the health status of the environment, and to predict the response of microbial communities to anthropogenic changes [10,11]. However, functions of microbial communities are largely ignored in biological monitoring methods [12]. A recent study recommended that the structural or functional prokaryotic variables (biodiversity, abundance and metabolism) should be included in future implementations of the EU Marine Strategy Framewok Directive 2008/56/EC (MSFD) [13].

Metagenomics has been successfully applied to investigate microbial diversity, adaptation, evolution, and function [14]. The profiling of microbial communities can be revealed by high-throughput sequencing of targeted PCR amplification [15]. For example, through 16S rRNA genes sequencing, the microbial community variation can provide important baseline understandings of the microbial ecology and health assessment of the marine ecosystems [16,17]. Metabolism of microbial communities can be investigated by metagenomics without any prior knowledge. The metagenomic results are important evidences to measure some specific ecological processes. For instance, the metagenomic analysis revealed the metabolic versatility of microorganisms and their roles in biogeochemical cycles including nitrogen, carbon, and sulfur cycles at the Yap Trench of the western Pacific [18]. Metagenomics was also applied to study the upper and core regions of oxygen minimum zones in Arabian Sea, and confirmed the genomic potentials of active nitrogen cycle [19]. The metagenomic results revealed that the metabolic capacity of microorganisms might be weaken by contamination in mangrove sediments and the increase of greenhouse gas emission might be induced [20]. Recently, combining of 16S rRNA amplicon sequencing and metagenomic shotgun analysis revealed that chemical pollutants severely affected microbial structures and functions in sediments [21]. Therefore, high-throughput sequencing of amplicons and metagenomic shotgun sequencing can provide new perspective of complexity of microbial communities and their functional trait [22].

Bohai Sea is located in Northeast China and has been influenced by anthropogenic factors in recent years [23,24]. Previous studies revealed that the pollution in Bohai Sea is serious in coastal areas and that situation can be worse if no protection procedures are implemented [25]. The mariculture activities of Bay scallops (*Argopecten irradians*) have been conducted for over 30 years, and is a major industry that supports the economy of Qinhuangdao, which is a coastal city of Bohai Sea [17]. Hydrargyrum, Cadmium, and Plumbum are the predominant metal pollutants commonly found in the Bohai Sea, and their concentration in some areas were 12 to 150 times higher than the background concentrations [25]. In addition, Cadmium in fish species from Qinhuangdao had higher concentrations than that from the Pearl River Estuary, Zhejiang and Haikou of China [26].

Mitigating the threat of human activities to marine environment is a big challenge, thus it is necessary to develop new methods to monitor and assess the impact of human activities on marine sediments and the surrounding seawater columns in the mariculture areas, which could help us understand the restoration potentials of the marine ecosystems[27]. Previous studies have characterized the bacterioplankton communities of natural seawaters in Bohai Sea, and investigated the archaeal diversity and community structures in this area [16,17]. However, the lack of understanding of the metabolic capability of microorganisms in the sediments and surrounding seawaters of the Qinhuangdao mariculture area hinders the prediction of the capability of the microorganisms in responding to changes of environmental conditions. To our knowledge, this would be the first study to characterize the microbial communities with their metabolic potentials in Qinhuangdao mariculature area.

In this study, we collected two surface sediments samples and two surrounding seawater samples in Qinhuangdao mariculture area. Sequencing of 16S rRNA gene amplicons was used to identify the microbial communities. Metagenomic analysis was carried out to reveal the metabolic potentials. The goal of this research was to: (1) investigate the compositions of microbial communities in the sediments and surrounding seawaters; (2) provide the yet unexplored representative characteristic of metabolic potentials in Qinhuangdao aquaculture area; (3) identify the differences of metabolic potentials and key genes associated microbial communities in sediments and seawaters. The results of this study could help us understand the microbial communities and their metabolic patterns in Qinhuangdao mariculture area.

## Materials and methods

### Samples collection and environmental parameters description

**Fig 1:**
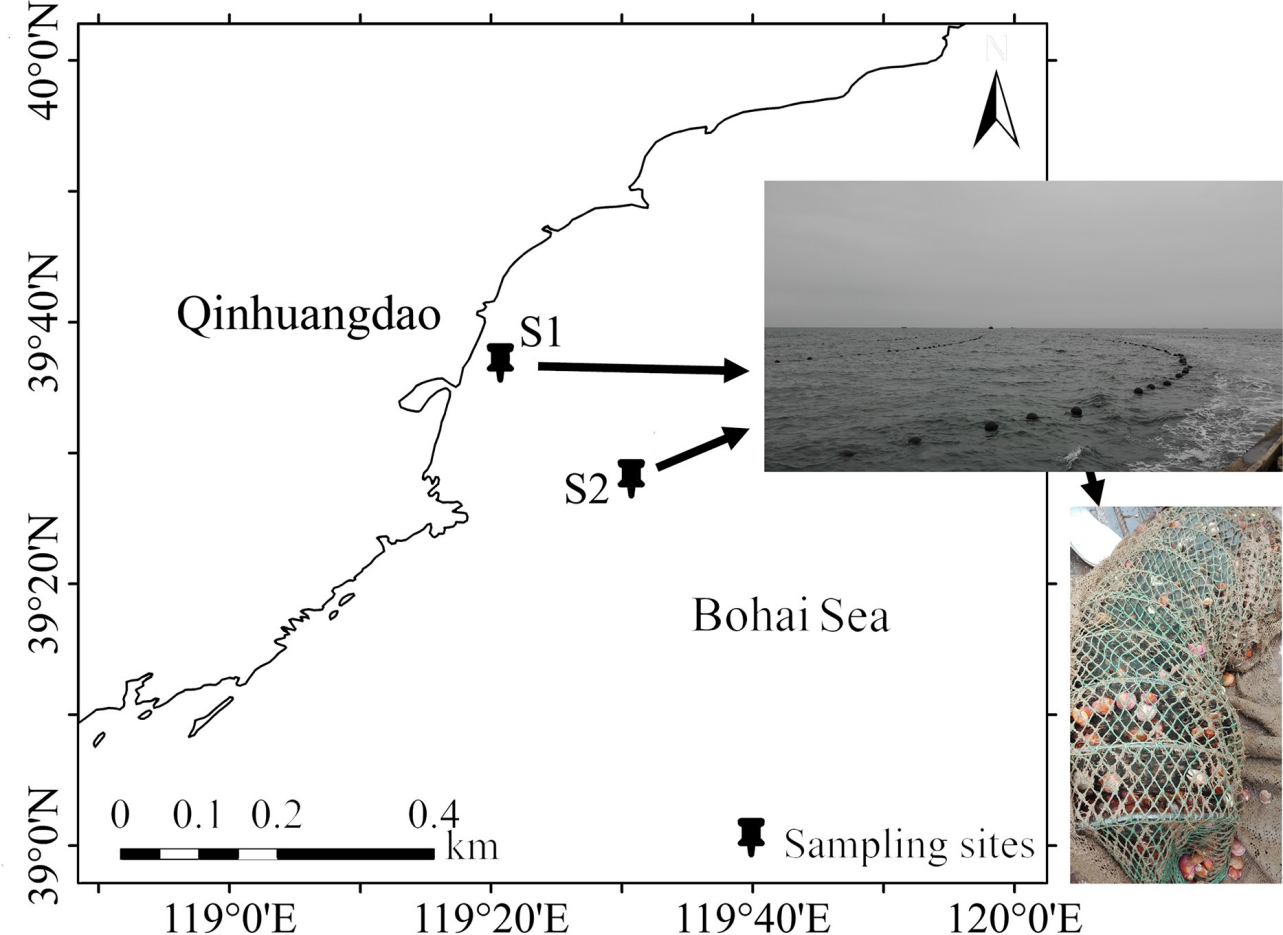
Map of study areas showing geographical locations, raft cultivations of *A*.*irradians*, and mesh cage used for cultivation. ArcGIS 10.1 software (http://www.esri.com/software/arcgis) was used to develop the map.

The field site access was approved by the departments of fishery administration. The seawaters were sampled on July 26, 2017 in two stations, S1 (39˚36’53” N, 119˚20’43” E) and S2 (39˚34’43” N, 119˚25’32”E) in Qinhuangdao mariculture area that farmed *A*. *irradians*, from 8 m and 10 m depth, respectively. (Fig 1). Seawaters were sampled in triplicate at each station, then homogeneously mixed prior to filtration, five litres of the pooled water were filtered through a 0.2 μm filter membrane (Millipore, Billerica, USA). Five grams of surface sediments were concomitantly collected at the S1 and S2 locations using a grab sampler made of stainless steel, and the sediment samples were named as S1S and S2S respectively. The sampled sediments were stored in sterile 5 ml storage tubes. All the filter membranes and sediments were stored at -80˚C for further analysis. Physicochemical properties of the seawater and sediment samples were determined. In detail, the values of parameters including temperature, pH, dissolved oxygen (DO), salinity, turbidity, electrical conductivity (EC), total dissolved solids (TDS), oxidation-reduction potential (ORP), total nitrogen (TN), total phosphorus (TP), total organic carbon (TOC) and total sulfur (TS) content in the seawater or sediments were determined (S1 Table).

### DNA extraction, amplicon sequencing and analysis

The MoBio PowerSoil^®^ DNA Isolation Kit (MO BIO Laboratories, USA) was used to extract the DNA from 0.2-μm filter membrane or 5 g of sediment. DNA concentration and quality were determined using NanoDrop spectrophotometer (ThermoScientific, Wilmington, DE, USA). The DNA extracts were used as the templates in PCR. The V4 variable region of the 16S rRNA genes was amplified by primers 16S V4-515F (5’-GTGCCAGCMGCCGCGGTAA-3’) and 16S V4-806R (5’-GGACTACHVGGGTWTCTAAT-3’) [28]. The 16S rRNA gene amplicons were prepared and subsequently sequenced with Illumina HiSeq high-throughput sequencing (2╳250 bp). Using the Qiime2 pipeline, effective tags were obtained through analyzing the sequencing output [29]. Operational Taxonomic Units (OTUs) were clustered by the effective tags at 97% sequence similarity. The analysis of taxonomic annotation was performed based on SILVA v132 reference database [30]. Relative abundance was represented by the percentage of each taxon in each sample to compare their microbial communities in phyla and class levels. The difference between the four communities was investigated by principal component analysis (PCA) using R software packages by considering the relative abundance of all microbial genera.

### Metagenomic shotgun sequencing and analysis

The DNA extraction method was the same and described above. DNA was used for library preparation and detection. Qualified libraries were sequenced using Illumina with the paired-end length of 2╳150 bp, and the final raw data was used for bioinformatic analysis. The raw data was filtered by trimming adapter sequences and low quality reads (quality value less than 38 ≥40bp, and ≥10% N containing reads). The clean reads were *de novo* assembled for each DNA sample independently using MEGAHIT (—presets meta-large).The unassembled reads in each sample were merged together and assembled [31]. ORFs were predicted from the contigs of each sample as well as the contigs from the merged assembly using the MetaGeneMark software. Predicted genes were subjected for de-redundancy analysis using CD-HIT. Clean reads were mapped to representative genes by Bowtie 2, the number of reads mapped to each gene were used for the calculation of gene abundance [32,33]. Genes of which reads number in each sample was no more than 2 were filtered, thus the unigenes were obtained and used for subsequent analysis. The predicted protein sequences were searched by BLASTP with e-value≤1╳10^−5^ in the Kyoto Encyclopedia of Genes and Genomes (KEGG) database [34]. The annotated genes were assigned into KEGG pathways. Based on the functional abundance of level 3 KEGG pathways, PCA was used to cluster the samples. According to PCA clustering results, Metastat statistical analysis and metabolic pathway comparison analysis were carried out to distinguish functional composition differences.

### Nucleotide sequence accession numbers

Metagenomic datasets are available online at NCBI, BioSample accessions: SAMN13744940, SAMN13744941, SAMN13744942, SAMN13744943 for Seawater1 (S1), Sediment1 (S1S), Seawater2 (S2), and Sediment2 (S2S) samples respectively. The 16S rRNA datasets are available online at NCBI, BioSample accessions: SAMN13752121, SAMN13752122, SAMN13752123, SAMN13752124 for (Seawater sample1) S1, (Sediment sample1) S1S, (Seawater sample2) S2, and (Sediment sample2) S2S samples respectively.

## Results

### Taxonomic characterization of the microbial communities

**Fig 2:**
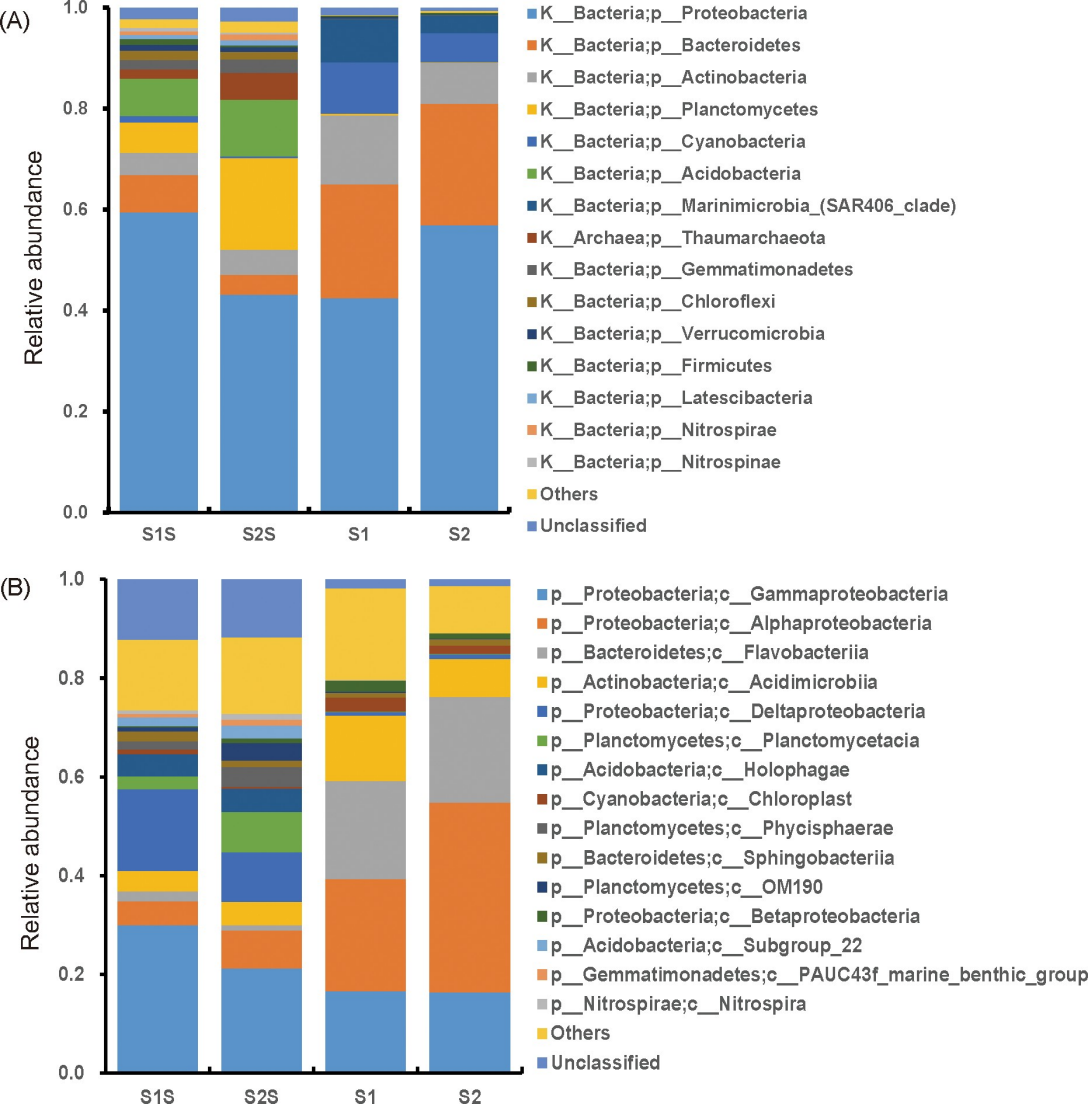
High-throughput sequencing of 16S rRNA gene revealing the relative abundance of phylum (A) and class (B) in the sediment (S1S and S2S) and seawater (S1 and S2) samples. The top 15 phyla or classes with the largest relative abundance were presented and the rest phyla or classes were set as others or unclassified.

Based on the sequencing of 16S rRNA genes, 61,799, 88,645, 91,113 and 86,122 clean reads were obtained from the S1S, S2S, S1 and S2 samples, respectively. A total of 4,784 OTUs were found in the sediment samples while only 860 OTUs were in the seawater samples. Specifically, the numbers of OTUs detected in each sample were 3,530, 4,271, 709, and 617 in S1S, S2S, S1, and S2 samples, respectively. The relative abundance of the phyla and classes in different samples are shown in Fig 2. Proteobacteria, Bacteroidetes, and Actinobacteria are the three most dominant phyla, accounting for 49.69%, 15.04%, and 8.08%, respectively (Fig 2A). At class level, the top three dominant classes were Gammaproteobacteria (20.35%), Alphaproteobacteria (19.36%), and Flavobacteriia (11.85%). Specifically, Gammaproteobacteria and Alphaproteobacteria are both the sub-divisions of Proteobacteria. Gammaproteobacteria was the dominant class of in sediments (25.6%) while the dominant class in seawaters was Alphaproteobacteria (30.5%) (Fig 2B).

### Functional profiles of microbial communities from KEGG functional analysis

After removing low quality reads, a total of 51 Gbp clean data was generated for the four samples, with 12.9 Gbp for S1, 12.3 Gbp for S2, 12.3 Gbp for S1S, and 13.2 Gbp for S2S. After assembling of the clean reads, there were 435,438 contigs with the average length of 974 bp. Venn diagram showed the number of identified genes and their distribution in sediments and seawaters (S1 Fig). There were 280,552 genes common for sediments and seawaters, while there were 666,483 and 1,024,221 genes for sediments and seawaters, respectively (S1 Fig). Functional properties of these genes were investigated using KEGG Orthology (KO). Of the identified protein-coding genes, 45.9% were assigned against KO database. In detail, the number ratios of genes involving in organismal systems, metabolism, human diseases, genetic information processing, environmental information processing, and cellular processes were 1.65%, 31.26%, 3.19%, 5.86%, 4.25%, and 3.80% (S2 Fig). Besides, the relative abundances of reads assigned to level 1 and level 2 KEGG pathways in the four samples were showed in S3 Fig.

### Differential analysis of microbial taxonomic profiles and metabolic pathways

**Fig 3:**
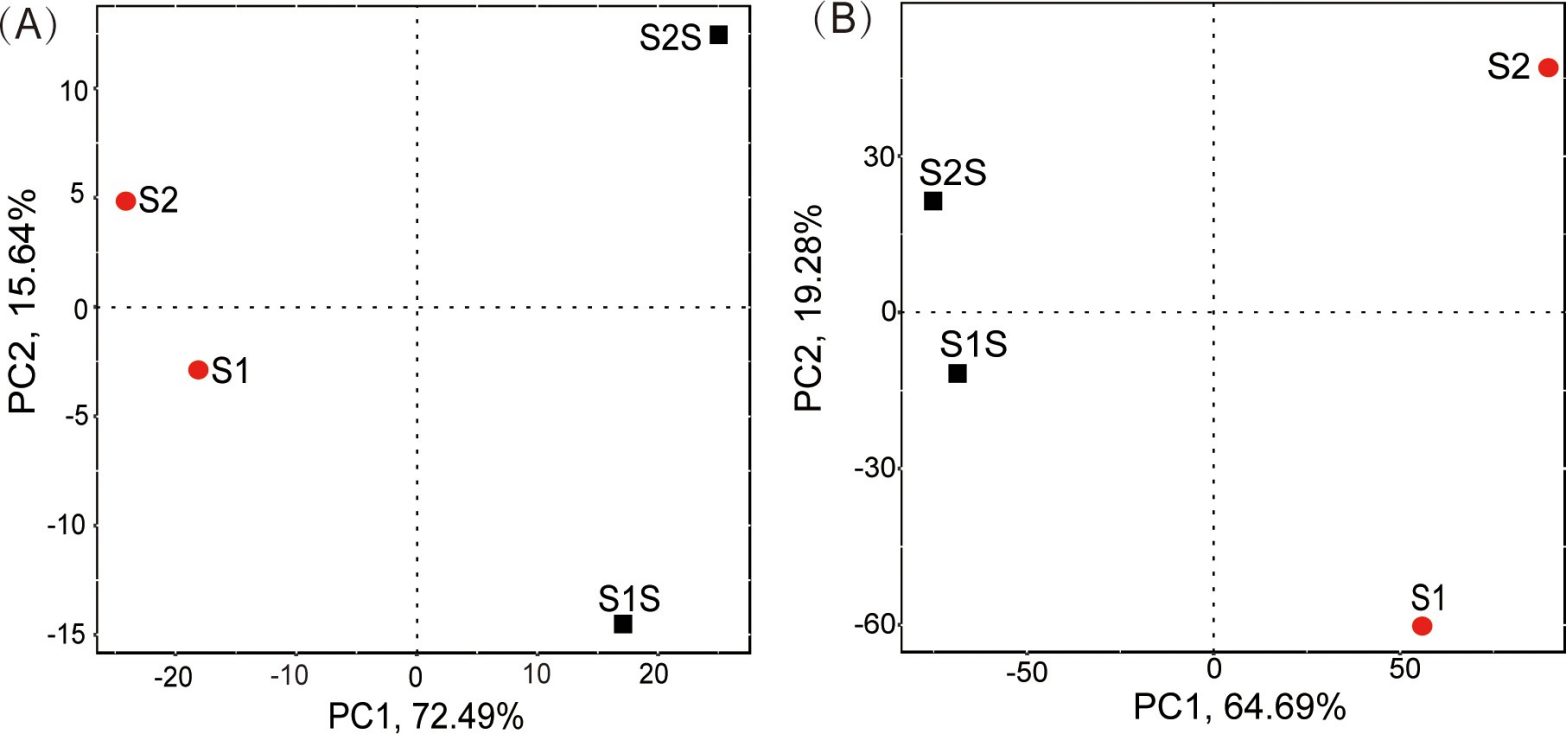
PCA analysis results based on (A) genus abundance from 16S rRNA gene amplicon sequencing and (B) functional abundance of level 3 KEGG pathways. The red and black dots are sediment and seawater samples, respectively. The abscissa and the ordinate represent the first principal component (PC1) and the second principal component (PC2) respectively. The percentage represents the contribution of the principal components to the samples difference.

PCA was used to statistically explore and visualize the difference between the different microbial communities (Fig 3). Two seawater samples and two sediment samples were clustered together based on the microbial compositions at the genus level (Fig 3A). PCA was also performed to cluster the metagenomic data based on their KEGG annotations. Obvious differential distributions of KEGG pathways between the sediments and seawaters were observed (Fig 3B). Therefore, there are not only differences in microbial taxonomic profile but also significant variations in the potential metabolic pathways.

### Top abundant pathways from KEGG functional analysis

**Fig 4:**
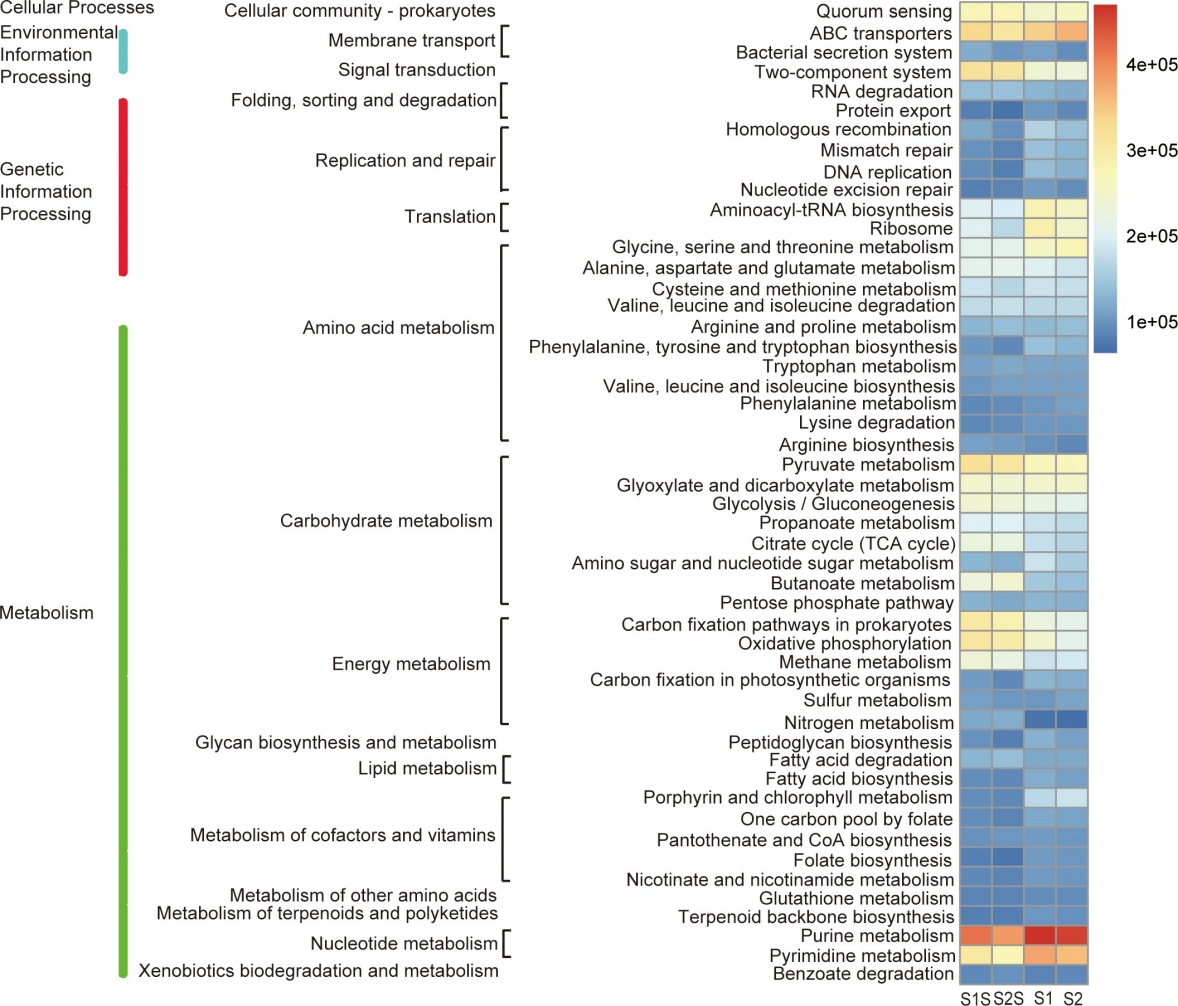
Heatmap profile showing the relative abundance of top 50 most abundant metabolic pathways in the sediment and seawater samples. The three columns of descriptions from left to right represent the level 1, level 2, and level 3 KEGG pathways.

The top three abundant pathways were purine metabolism, ABC transporters and pyrimidine metabolism. Furthermore, the purine metabolism and pyrimidine metabolism belong to nucleotide metabolism. ABC transporters belong to membrane transport pathway (Fig 4).

#### Purine metabolism

A total of 208 genes were involved in purine metabolism in the sediment and seawater samples. The highest abundance gene was *nrdAE* that encoding ribonucleoside-diphosphate reductase alpha chain. In addition, *rpoB*, *rpoC*, *PFAS*, *polA* and *dnaE* that encoding DNA-directed RNA polymerase subunit beta, DNA-directed RNA polymerase subunit beta', phosphoribosylformylglycinamidine synthase, DNA polymerase I, and DNA polymerase III subunit alpha also exhibited high abundance.

#### ABC transporters

323 genes annotated as ABC transporters were retrieved in the four metagenomes. Of these genes, genes (*livF*, *livG*, *livK*, *livM*, and *livH*), which belonged to branched-chain amino acid transport system, showed the highest abundances.

#### Pyrimidine metabolism

A total of 143 genes involved in pyrimidine metabolism were identified. Similar to purine metabolism, *nrdAE* was also the highest abundant gene, followed by *rpoC*, *rpoB*, *CPA2* (encoding carbamoyl-phosphate synthase large subunit), *dnaE*, and *polA*.

### Differentially abundant pathways from KEGG functional analysis

**Fig 5:**
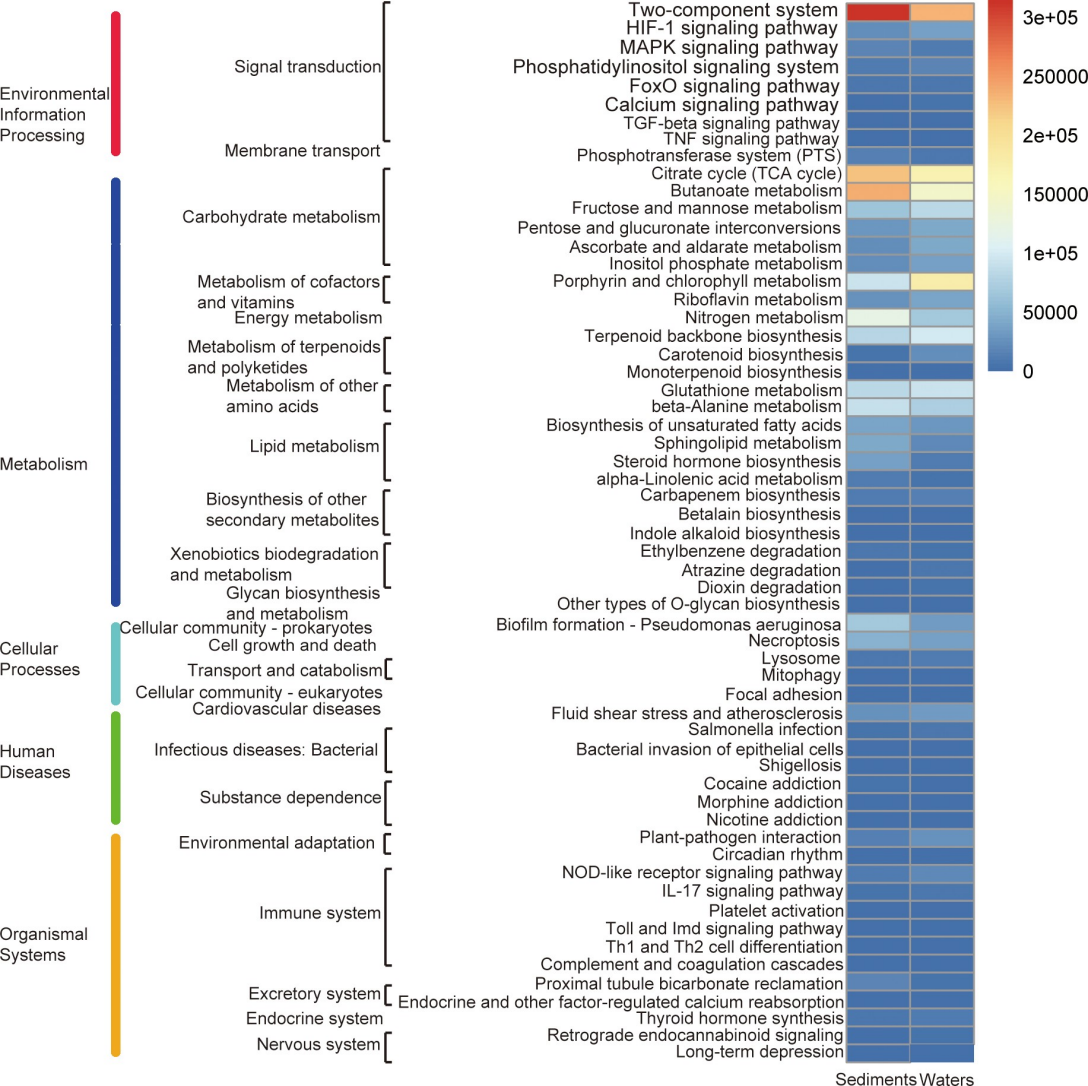
The relative abundance of top 50 most differentially abundant metabolic pathways in the sediment and seawater samples. The three columns of descriptions from left to right represent the level 1, level 2, and level 3 KEGG pathways.

Further analysis showed that two-component system, citrate cycle (TCA cycle), and nitrogen metabolism were identified as differentially abundant pathways (Fig 5).

#### Two-component system

**Fig 6:**
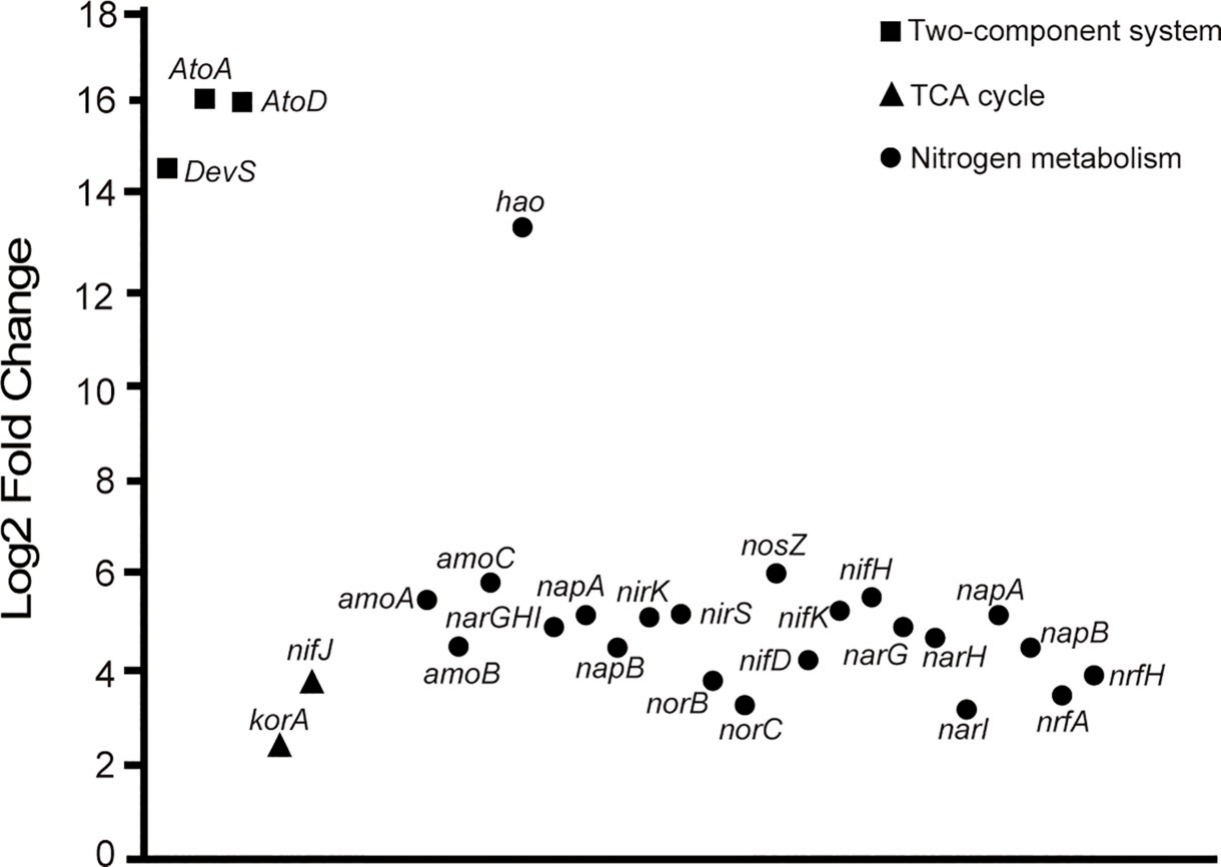
Fold changes of selected genes that were more abundant in sediments than in seawaters. Genes were selected from two-component system, TCA cycle, and nitrogen metabolism.

A total of 295 genes were present in the two-component system. Of these 295 genes, 182 genes exhibited higher abundance in sediments than in seawaters, while 113 genes exhibited higher abundance in seawaters than in sediments. Notably, genes including *DevS*, *AtoA*, and *AtoD* that encode sensor histidine kinase DevS, acetoacetate CoA-transferase beta subunit, and acetoacetate CoA-transferase alpha subunit were only identified in sediment samples (Fig 6).

#### TCA cycle

A total of 59 genes involved in TCA cycle were identified for all the samples. 36 genes exhibited higher abundance in sediments than in seawaters, while 23 genes exhibited higher abundance in seawaters than in sediments. Overall, TCA cycle exhibited high abundance in sediments compared with that in seawaters. The genes *nifJ* and *korA* that encoding pyruvate-ferredoxin/flavodoxin oxidoreductase and 2-oxoglutarate/2-oxoacid ferredoxin oxidoreductase subunit alpha were more abundant in the sediments significantly (*P*<0.05) (Fig 6).

#### Nitrogen metabolism

Microbial communities in both sediments and seawaters possessed diverse metabolic genes involved in nitrogen metabolism (Fig 4, Fig 5). 36 genes were more abundant in sediments than in seawaters. The predicted nitrogen metabolism pathway was summarized in S4 Fig. For instance, genes ammonia monooxygenase (*amoA*, *amoB*, and *amoC*) were more abundant in the sediments than in the seawaters. The sequences annotated as “*hao”* were only found in sediment metagenomes. The marker genes including *narGHI* or *napAB*, *norBC*, *nirK* or *nirS*, *nosZ* encode the denitrification-related enzymes including nitrate reductase, nitric oxide reductase, nitrite reductase, and nitrous oxide reductase. These genes were all more abundant in sediments than in seawaters. Moreover, there were more abundant genes involved in nitrogen fixation (*nifDKH*) and in dissimilatory nitrate reduction to ammonium (DNRA) (*narGHI*, *napAB*, *nirBD*, and *nrfAH*) in sediments than in seawaters (Fig 6).

### Identification of cadmium-resistant genes

In particular, the metagenomics revealed several cadmium-resistant genes. These genes include *czcA*, *czcB*, *czcC*, *czcD*, *cadC*, and *zipB* coding for czcA (cobalt-zinc-cadmium resistance protein), czcB (membrane fusion protein), czcC (outer membrane protein), czcD (cobalt-zinc-cadmium efflux system protein), cadC (lead/cadmium/zinc/bismuth-responsive transcriptional repressor), and zipB (zinc and cadmium transporter). The relative abundances of cadmium-resistant genes in sediments and seawaters were summarized in S3 Table.

## Discussion

The present study is based on the comparative metagenomic evaluation of sediments and surrounding seawaters in Qinghuangdao mariculture area. The identification of microbial community compositions and their metabolic pathways could provide valuable insights into the biogeochemical process in Qinhuangdao coastal mariculture area.

According to the sequencing of 16S rRNA genes, high abundance of Proteobacteria is a common feature in both sediments and seawaters, and this phylum has been characterized to actively participate in biogeochemical processes of lake or marine ecosystems [35,36]. Previous study had revealed that Proteobacteria showed the highest relative abundance in sediments of the Bohai Sea, Yellow Sea and South China Sea [37]. Bacteroidetes and Actinobacteria were also the major phyla in cooccurrence with Proteobacteria, which are the typical heterotrophic bacteria playing roles in degrading marine organic nitrogen [37]. Similar to our results, many researches have reported that Proteobacteria (especially Alphaproteobacteria and Gammaproteobacteria), Bacteroidetes and Actinobacteria are the most important components of the offshore microbiome [38–42]. On class level, Gammaproteobacteria and Alphaproteobacteria were the most predominant class in sediments and seawaters, respectively. The result is consistent with previous findings that Gammaproteobacteria is an important class that has universal distribution in marine sediments [43] while Alphaproteobacteria dominates in the temperate coastal marine waters [44].

The sediment samples clustered distinctively from seawater samples and displayed a higher microbial diversity than the seawater samples. The metagenomic data also suggested that the sediment samples obtained from two different locations were more similar, while the seawater samples from two different locations clustered together. Therefore, the habitat substrates are likely the main factors shaping the microbial community characteristics not only at taxonomic level, but also at metabolic level. Studies have found that sediment contains more nutrients than seawater column because of the reservoirs of absorbed nutrients and high-molecular-weight organic nitrogen, and protects microorganisms from sunlight and predation. These advantages lead to sediments contain greater microbial diversity and higher microbial abundance than in the seawater columns [45,46]. Rich nutrient sediments in aquaculture have the necessary conditions for the proliferation of entire microbial communities and may be a source of new genes [47].

The microbial communities can mediate diverse metabolic processes that might be generally underrepresented. It is noteworthy that purine metabolism and pyrimidine metabolism were the most active pathways in all the communities. Ribonucleoside diphosphate reductase has been considered to be a protein that is rigorously required for elongation of DNA replication, and has a key role in regulating DNA synthesis [48]. The *dnaE* gene encodes the catalytic subunit of DNA polymerase III that is the main DNA replicative polymerase. The *polA* gene encodes DNA polymerase I that plays a role in excision-repair processes to process Okazaki fragments and fill gaps [49]. The genes *rpoB* and *rpoC* encode the major catalytic subunits of RNA polymerase in bacteria [50]. Phosphoribosyl formylglycinamidine synthase is involved in the purine biosynthetic pathway, which is necessary for nucleic acid synthesis and formation of ATP. The large subunit of arginine-specific carbamoyl phosphate synthetase was encoded by CPA2 gene and is involved in arginine biosynthesis [51]. All the highly abundant genes reported here suggested their important roles in synthesis of nucleic acid and amino acid. Result also indicated that ATP-binding cassette (ABC) transporters of microbial communities were active in this mariculture area, which couple ATP hydrolysis to actively transport of a wide variety of nutrients (e.g., some sugars, amino acids, and vitamins) [52]. The most conspicuous signature of the ABC transporters is the very high abundance of *livF*, *livG*, *livK*, *livM*, and *livH* genes that encode branched-chain amino acid transport ABC transport system. The function of this system is absorbing and transporting exogenous branched-chain amino acids by cells [53]. As one of an organism’s primary interfaces with the environment, the microorganisms probably heavily invested in the ABC transporters to efficiently sense and response to the environmental changes [54].

Two-component systems play important roles in adapting changing environmental conditions for microorganisms. Through two-component systems, microorganisms can modify cellular physiological activities including catalyzing reactions, initiating gene expression, or modifying protein-protein interactions [55]. DevS has been found to be a sensor of hypoxia, CO, and NO, and catalyze a reaction of DevR with ATP to produce phosphor-DevR [56]. The genes involved in metabolism of short-chain fatty acid constitute ato system. *AtoA* and *AtoD* supported the pathway of degradation of short-chain fatty acid in the sediments and eventually produced ATP by TCA cycle [57]. The high abundance of genes in TCA cycle in sediments compared to in seawaters might be indicative of the standard pathway for heterotrophic microorganisms to synthesize ATP and carry out a wider metabolic network contributing to other aspects of metabolism [58,59]. Notably, the gene coding for PFO (*nifJ*) as a part required for nitrogenase expression is apparently present in the TCA cycle [60]. Nitrogen cycle is critical for removing excess nutrients from marine ecosystem species. Research has revealed that the microbial communities in the upper Mississippi River sediment had large genomic potential for nitrogen cycling pathway regardless of mussel assemblages [61]. As key enzymes for nitrification, ammonia monooxygenase (*amoA*, *amoB*, and *amoC*) and hydroxylamine dehydrogenase (*hao*) co-catalyze the oxidation of ammonia to nitrite. The presence of *amoA*, *amoB*, *amoC* and *hao* in the sediments suggested that the process hydroxylamine might be an important intermediate. In that process, ammonia was used as an energy source and then the hydroxylamine was catalyzed to nitrite [62]. Enzymes including nitrate reductase, nitrite reductase, nitric oxide reductase, and nitrous oxide reductase are encoded by *narGHI* or *napAB*, *nirK* or *nirS*, *norBC*, and *nosZ* respectively. All the enzymes participate in the denitrification processes including the reduction of NO_3_^-^ to NO_2_^-^, NO, N_2_O, and N_2_. The more abundance of these marker genes in the sediments suggests the elevated capacity of denitrification in sediments.

Cadmium is the most common heavy metal and is easily detected in shellfish farming environments worldwide. The existence of some genes with specific roles has important ecological significance. The presence of cadmium-resistant genes suggests the ability of microorganisms in this environment to resist the toxicity of cadmium. Proteins including czcA, czcB, czcC, and czcD are encoded by *czc* operon [63] which can pump Zn (Ⅱ), Cd (Ⅱ), and Co (Ⅱ) from cells, and constitute the best understood genetic mechanism of bacterial metal resistance [64]. The *cadC* gene encodes Cd(Ⅱ)/Pb(Ⅱ)-specific ATPase efflux pump and regulates *cad* cadmium resistance operon expression [65]. ZIPB protein, the Zrt-, Irt-like protein (ZIP) homolog which can transport a broad spectrum of metal ions including Cd^2+^, Zn^2+^, Cu^2+^, and Mn^2+^ [66]. All the genes above related to cadmium-resistance help assessing the functional potential of cadmium tolerance or resistance associated with the microbial community. In addition, ABC transporters can also have an vital role in detoxification of heavy metal, and resistance to the contamination of cadmium [67,68].

As far as we know, this is the first study to report the molecular inner operation mechanism of sediments and surrounding seawaters in the Qinghuangdao mariculture area. This study is a starting point to monitor the microbial communities and their metabolisms in Qinhuangdao mariculture area, especially those impacted by aquaculture activities. Although data provided here only reveals the metabolic potential of microbial community, it provides the basis for determining the actual metabolic activities of the microorganisms in the future.

## Conclusions

In summary, this study provides insights into molecular characteristics of the microbial community compositions and their metabolic potentials. From the 16S rRNA gene amplicon dataset, various levels of taxonomic diversity in the microbial communities could be outlined. Metagenomic data showed the key metabolic pathways together with key genes. This study leads to the findings as the follows: (1) Proteobacteria was the overwhelming phylum in both sediments and waters, and Gammaproteobacteria and Alphaproteobacteria were the predominate classes. (2) Habitat type (i.e., sediments and seawaters) not the locations might be the reason for the variation in the microbiome in the Qinhuangdao mariculture area. (3) The microbial communities in Qinhuangdao mariculture area were mainly predicted to be active on the purine metabolism, ABC transporters and pyrimidine metabolism. (4) Two component system, TCA cycle, and nitrogen metabolism were the more abundant pathways in the sediments than in the seawaters. (5) cadmium-resistant genes and ABC transporters might play roles in resisting the toxicity of cadmium. Together, these results evidenced a broad microbial diversity with versatile metabolic pathways for the microbial communities in Qinhuangdao mariculture area.

## Supporting information

S1 Fig Metagenomic sequencing revealing the differences of gene numbers in seawaters and sediments. (TIF)

S2 Fig Numbers of genes annotated for KEGG database. The number on the bar chart represents the number of genes on the annotation. Another axis is the functional annotation information in KEGG.
(TIF)

S3 Fig The relative abundances of reads assigned to KEGG pathways in each sample. The number on the bar chart represents the percentage of reads annotated to each KEGG pathway.
(TIF)

S4 Fig Schematic of the predicted nitrogen metabolism pathway. Enzyme commission number and name of gene product are shown in the boxes. Genes only found in sediments are labeled with asterisk. Enzyme commission number is shown in the boxes.
(TIF)

S1 Table The main physical and chemical parameters of sampling sites and the sequencing information of samples. (PDF)

S2 Table The relative abundance of genes in level 1 pathway in different samples. (PDF)

S3 Table The relative abundance of cadmium-resistant genes in sediments and seawaters. (PDF)
