# Supplementary figures and images for: Comparative metagenomics reveals the microbial diversity and metabolic potentials in the sediments and surrounding seawaters of Qinhuangdao mariculture area
Source: PLoS One. 2020 Jun 4;15(6):e0234128. doi: 10.1371/journal.pone.0234128 (PMC7272022; doi:10.1371/journal.pone.0234128)

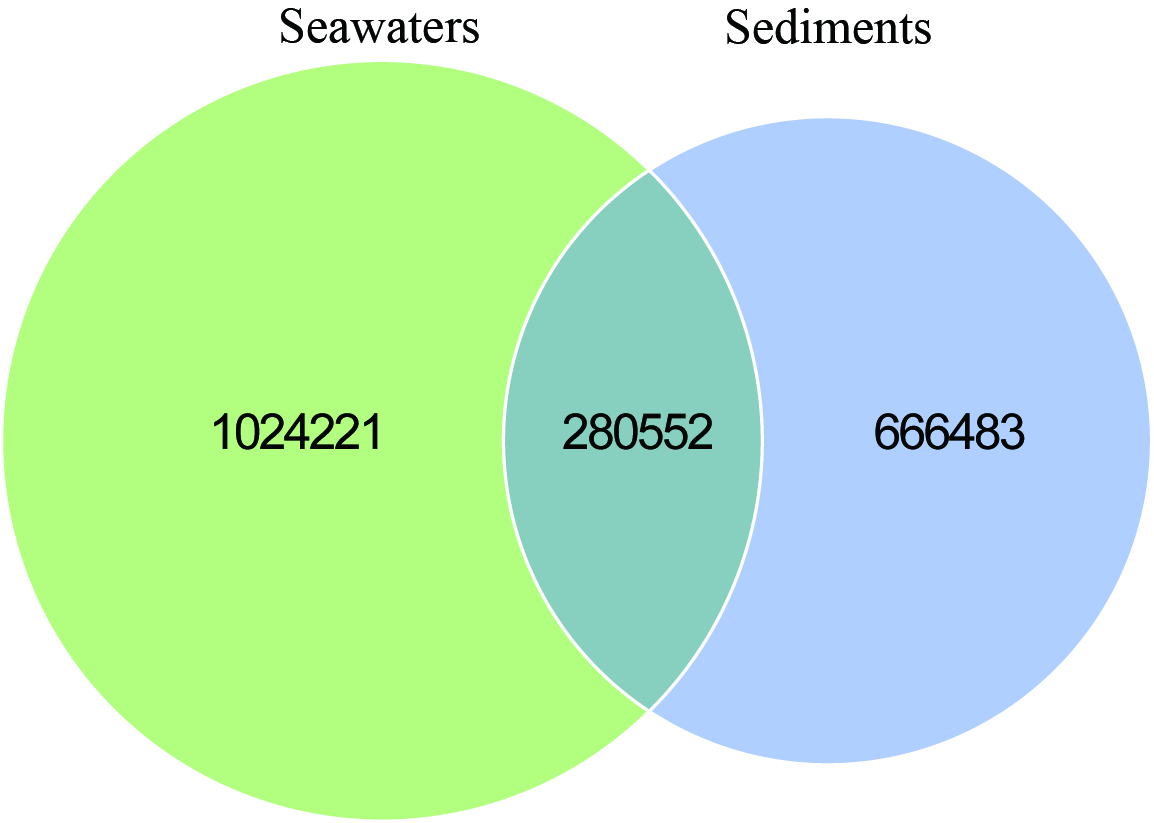

Supplement: S1 Fig — (TIF) [file pone.0234128.s001.tif]

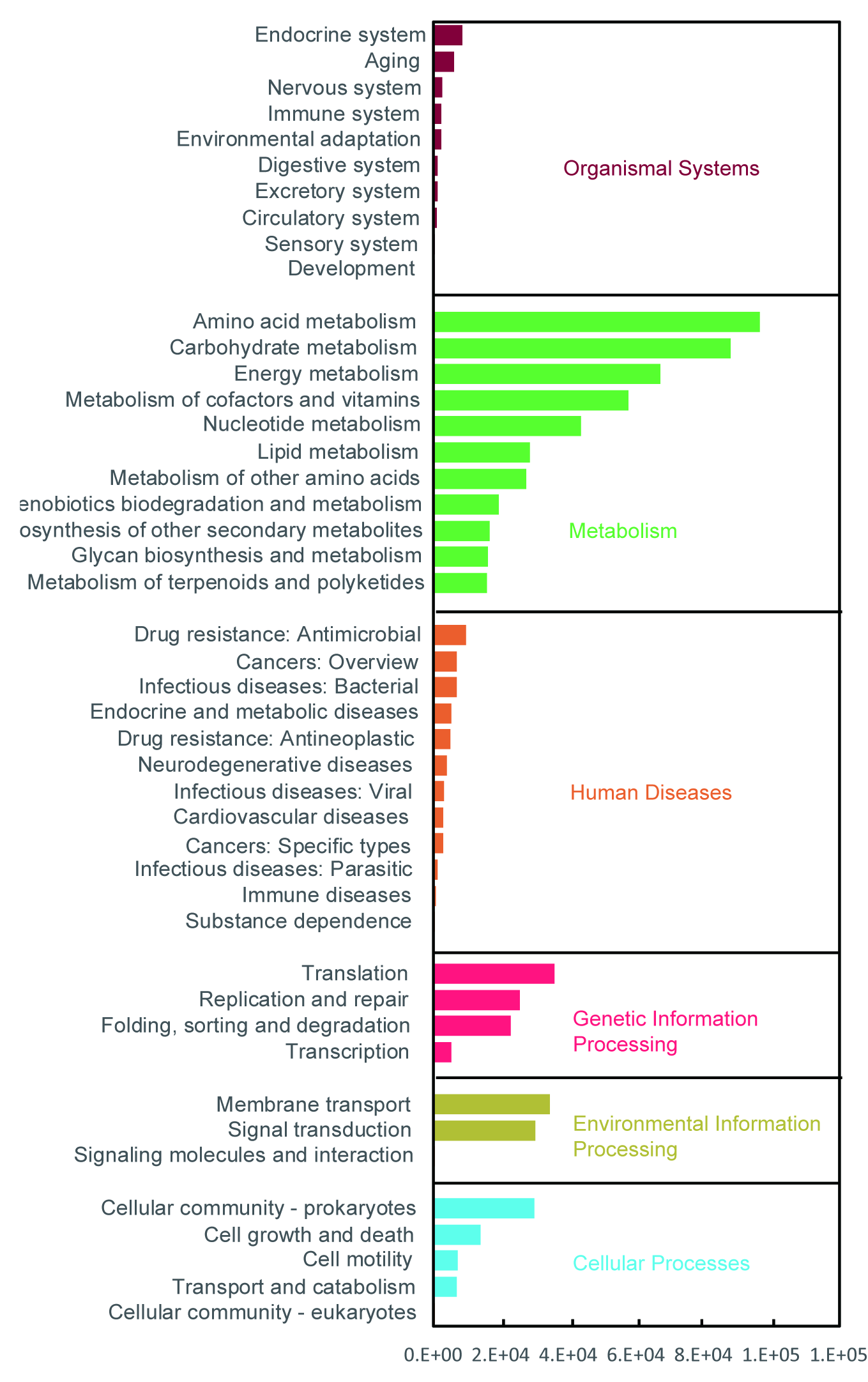

Supplement: S2 Fig — The number on the bar chart represents the number of genes on the annotation. Another axis is the functional annotation information in KEGG. (TIF) [file pone.0234128.s002.tif]

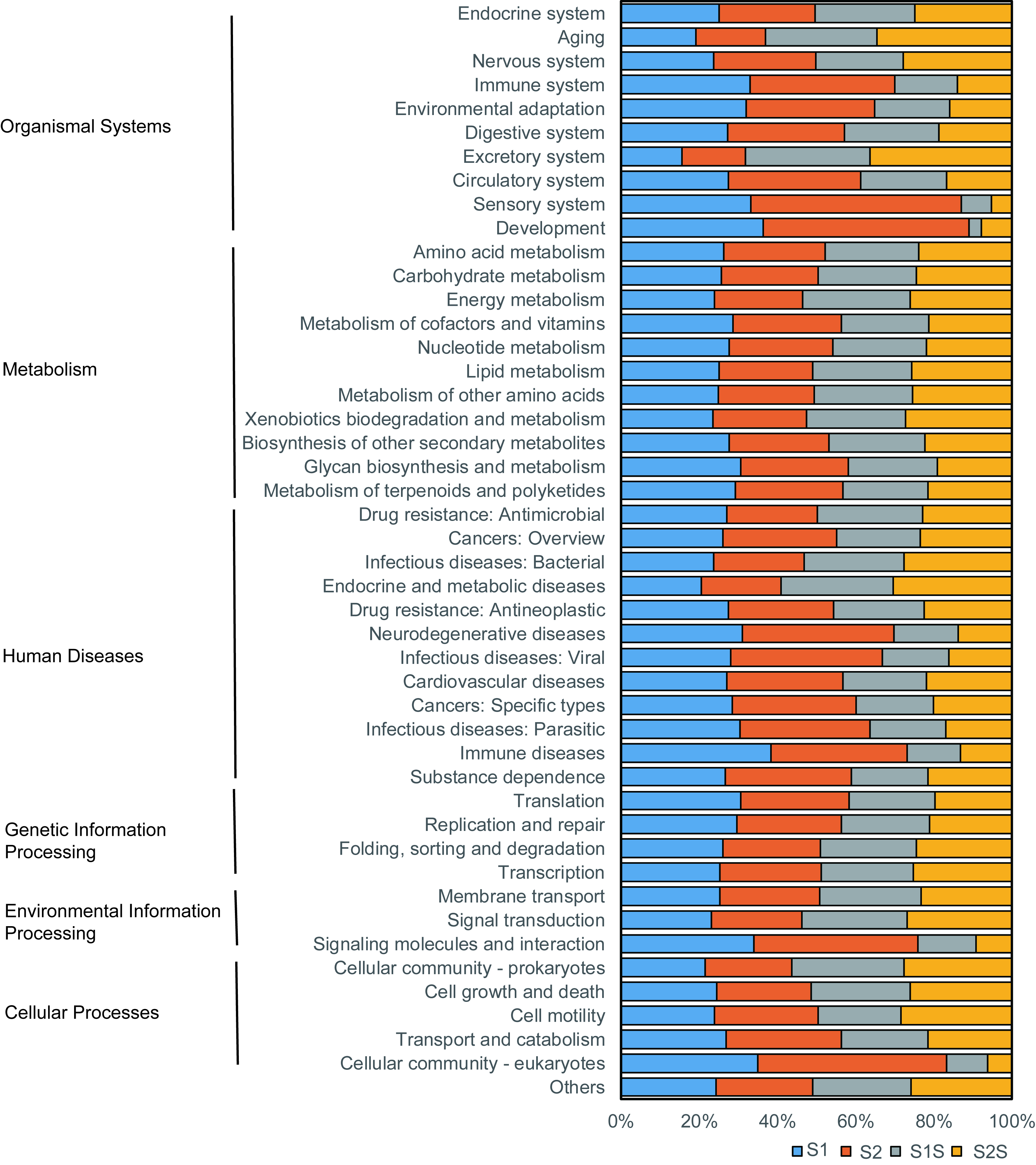

Supplement: S3 Fig — The number on the bar chart represents the percentage of reads annotated to each KEGG pathway. (TIF) [file pone.0234128.s003.tif]

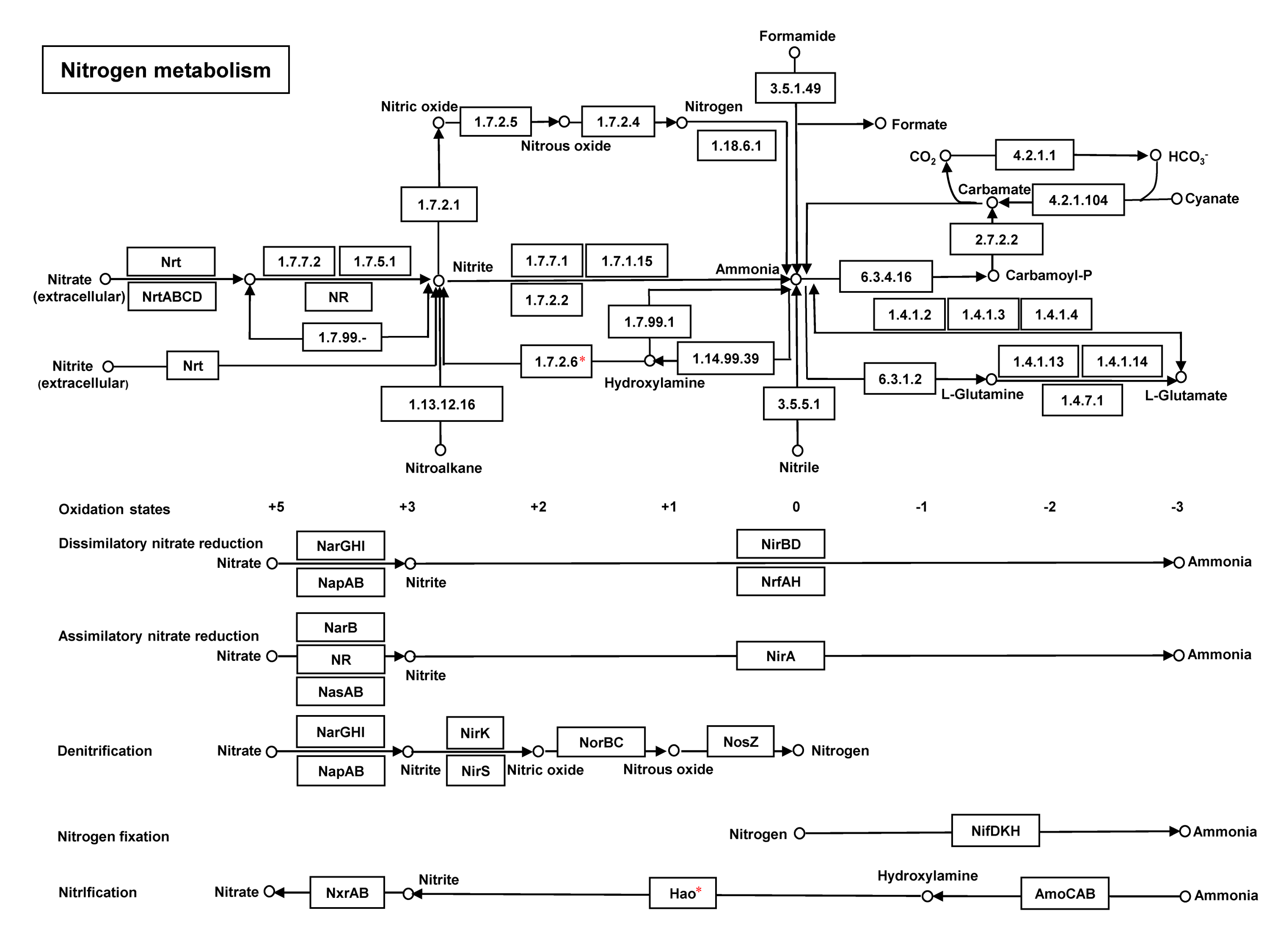

Supplement: S4 Fig — Enzyme commission number and name of gene product are shown in the boxes. Genes only found in sediments are labeled with asterisk. Enzyme commission number is shown in the boxes. (TIF) [file pone.0234128.s004.tif]
