# Supplementary material for: Comparative metagenomics reveals the microbial diversity and metabolic potentials in the sediments and surrounding seawaters of Qinhuangdao mariculture area
Source: PLoS One. 2020 Jun 4;15(6):e0234128. doi: 10.1371/journal.pone.0234128 (PMC7272022; doi:10.1371/journal.pone.0234128)
Supplement: S1 Table — (PDF) [file pone.0234128.s005.pdf]

Sample ID	S1S	S2S	S1	S2
**Temperature (℃)**	NA	NA	26.20	25.50
**pH**	8.41	8.25	6.71	6.97
**DO (mg/L)**	NA	NA	4.33	3.82
**Salinity (‰)**	NA	NA	31.80	32.50
**Turbidity (NTU)**	NA	NA	6.74	5.86
**EC (μS/cm)**	NA	NA	50.80	51.50
**TDS (mg/L)**	NA	NA	31.80	32.20
**ORP (mv)**	NA	NA	252.00	196.00
**TN (mg/L)**	0.10	0.10	5.15	4.57
**TP (mg/L)**	0.01	0.01	0.04	0.03
**TOC (mg/L)**	0.13	0.08	2.00	1.60
**TS (mg/kg)**	87.60	45.50	NA	NA
**Raw data (Mbp)**	12,329.28	13,195.41	12,967.57	12,330.96
**Clean data (Mbp)**	12,288.74	13,158.90	12,944.75	12,311.81
