# Supplementary material for: Comparative metagenomics reveals the microbial diversity and metabolic potentials in the sediments and surrounding seawaters of Qinhuangdao mariculture area
Source: PLoS One. 2020 Jun 4;15(6):e0234128. doi: 10.1371/journal.pone.0234128 (PMC7272022; doi:10.1371/journal.pone.0234128)
Supplement: S2 Table — (PDF) [file pone.0234128.s006.pdf]

KO_Pathway_Level1	S1S (%)	S2S (%)	S1 (%)	S2 (%)	Total (%)
Cellular processes	2.53	2.62	2.79	2.80	2.69
Environmental Information Processing	3.35	3.37	3.62	3.42	3.44
Genetic Information Processing	5.74	5.27	4.54	4.27	4.96
Human Diseases	1.90	1.85	1.75	1.69	1.80
Metabolism	18.69	17.92	17.06	16.33	17.50
Organismal Systems	1.02	1.01	1.14	1.21	1.09
Others	66.76	67.97	69.09	70.28	68.52
