# Supplementary material for: Comparative metagenomics reveals the microbial diversity and metabolic potentials in the sediments and surrounding seawaters of Qinhuangdao mariculture area
Source: PLoS One. 2020 Jun 4;15(6):e0234128. doi: 10.1371/journal.pone.0234128 (PMC7272022; doi:10.1371/journal.pone.0234128)
Supplement: S3 Table — (PDF) [file pone.0234128.s007.pdf]

Genes	sediments	seawaters	Description
czcA	2955.927	2136.484	cobalt-zinc-cadmium resistance protein CzcA
czcB	1018.055	916.0295	membrane fusion protein, cobalt-zinc-cadmium efflux system
cadC	600.2739	159.6633	lead/cadmium/zinc/bismuth-responsive transcriptional repressor
czcD	2384.147	935.5844	cobalt-zinc-cadmium efflux system protein
zipB	283.1985	928.4143	zinc and cadmium transporter
cacC	927.8136	629.2725	outer membrane protein, cobalt-zinc-cadmium efflux system
